# Development of imatinibmesylate-induced interstitial lung disease 2 weeks after discontinuation of the treatment: a case report

**DOI:** 10.1186/2049-6958-7-48

**Published:** 2012-11-23

**Authors:** Shota Nakashima, Tomoyuki Kakugawa, Hiroko Motomura, Katsuji Hirano, Eisuke Sasaki, Yasuhiro Nagata, Akitoshi Kinoshita, Noriho Sakamoto, Yuji Ishimatsu, Hiroshi Mukae, Shigeru Kohno

**Affiliations:** 1 Second Department of Internal Medicine, Nagasaki University School of Medicine, Nagasaki, Japan; 2 National Hospital Organization Nagasaki Medical Center, Nagasaki, Japan; 3 Nagasaki Prefecture Shimabara Hospital, Nagasaki, Japan; 4 University of Occupational and Environmental Health, Kitakyushu, Japan

**Keywords:** Drug-induced interstitial lung disease, Drug-induced lung injury, Drug induced pneumonitis, Drug lymphocyte-stimulating test, Imatinibmesylate

## Abstract

**Background:**

Imatinibmesylate (imatinib) is a small molecule tyrosine kinase inhibitor administered to patients with chronic myelogenous leukemia and gastrointestinal stromal tumor. Although imatinib-associated interstitial lung disease is uncommon, a few cases have been reported so far. However, in all these cases interstitial lung disease developed during the use of imatinib. The present case is the first report of imatinib-induced interstitial lung disease developing after discontinuation of the drug.

**Case presentation:**

A 51-year-old woman was administered oral imatinib for gastrointestinal stromal tumor. Ten weeks later, imatinib was discontinued because of facial edema. On this occasion, chest radiography showed no abnormal findings. However, 2 weeks after discontinuation of imatinib, she developed fever, dry cough, and dyspnea. Chest radiography and computed tomography showed diffuse interstitial infiltrates in both lungs. Examination of bronchoalveolar lavage fluid showed an increased proportion of lymphocytes. Imatinib-induced interstitial lung disease was suspected, because no other cause was evident. After administration of corticosteroids, her clinical condition and chest radiographic findings improved.

**Conclusion:**

We report a unique case of imatinib-induced interstitial lung disease that developed 2 weeks after discontinuation of the drug. Physicians should consider occurrence of imatinib-induced interstitial lung disease even after discontinuation of the drug.

## Background

Imatinibmesylate is a tyrosine kinase inhibitor (TKI) with activity against platelet-derived growth factor receptors (PDGFR-α and -β), discoidin domain receptors (DDR1 and DDR2), c-kit, and c-Abl. It is administered to patients with chronic myelogenous leukemia and gastrointestinal stromal tumor (GIST) [1,2].

Despite the encouraging data from cellular and animal models of lung fibrosis and the increasing use of imatinib to treat other inflammatory and fibrotic diseases such as scleroderma [3] and pulmonary arterial hypertension [4], few cases of imatinib-associated interstitial lung disease (ILD) have been reported so far [5] (and references therein). In all of these reported cases, ILD developed during the use of imatinib.

The present case is the first report about imatinib-induced ILD that developed after discontinuation of the drug.

## Case presentation

**Figure 1:**
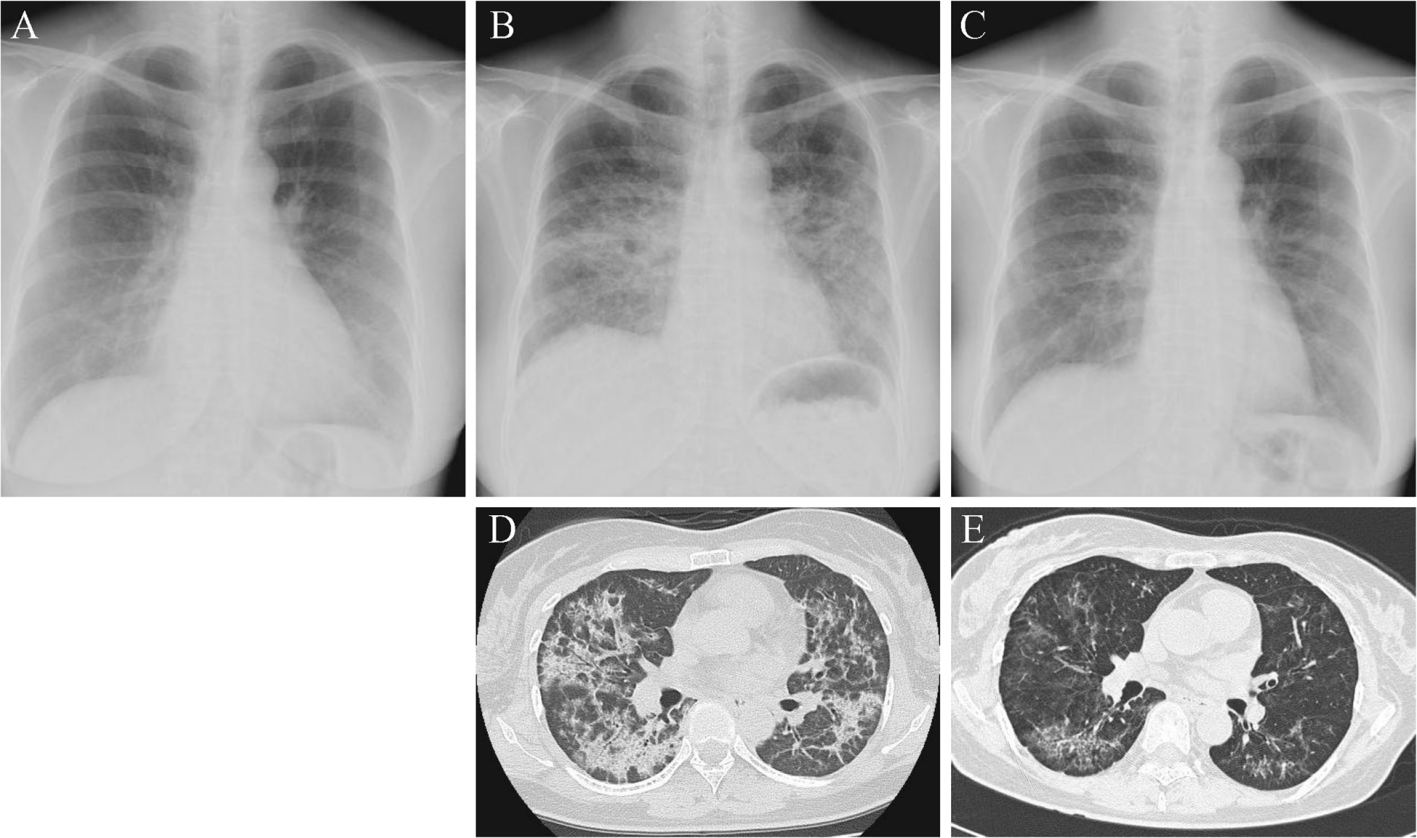
**Images of the patient’s lungs.** Chest radiography performed on the day imatinib was discontinued (**A**) showed no abnormal findings. Chest radiography on admission (**B**) revealed bilateral reticular shadows in both mid and lower lung fields. Chest high-resolution computed tomography (HRCT) scans obtained on admission (**D**) revealed diffuse interstitial infiltrates in both lungs, predominantly along the bronchovascular bundles extending to subpleural regions. Two months after corticosteroid therapy, chest radiography (**C**) and HRCT scans (**E**) indicated remarkable improvement.

A 51-year-old Japanese woman with no medical history was administered oral imatinib 400 mg daily for GIST. Ten weeks later, imatinib was discontinued because of facial edema. On this occasion, chest radiography showed no abnormal findings (Figure 1A). However, 2 weeks after discontinuation of imatinib treatment, she developed fever, dry cough, and dyspnea on exertion. Because her symptoms had remained unchanged for a month, she was referred to the respiratory medicine department in our hospital. She had taken no other drugs or alternative medicines.

The patient was a non-smoker. On admission, her oxygen saturation was 94% in room air, and body temperature was 36.2°C. Respiratory sounds were normal. Neither facial nor leg edema was observed. Arterial blood gas analysis in room air revealed a pH of 7.401; PaO_2_, 80.7 torr; and PaCO_2_, 40.1 torr. White blood cell count was 8,600/mm^3^ with 80.1% neutrophils, 11.8% lymphocytes, 5.4% monocytes, 2.6% eosinophils, and 0.1% basophiles. C-reactive protein was negative (<0.30 mg/dL). Levels of lactate dehydrogenase activity were slightly elevated (273 IU/L). Serum Krebs von den Lungen-6 (KL-6) and surfactant protein (SP)-D levels were normal (477 U/mL and 97.9 ng/mL, respectively). Anti-nuclear antibody and other autoantibodies to specific antigens were all negative. Pneumococcal and *Legionella* urinary antigens were negative. Cultures of blood and sputum to detect bacteria, fungi, and mycobacteria were all negative. Results of serological tests for *Mycoplasma pneumoniae* and *Chlamydophilapneumoniae* were negative; test results for β-d-glucan and cytomegalovirus antigen were also negative. The result of a drug lymphocyte-stimulating test (DLST) against imatinib was negative (Table 1).

**Table 1:** Laboratory data on admission

**Hematology**		
	WBC	8.6 × 10^3^/mm^3^
	Neutrophils	80.1%
	Lymphocytes	11.8%
	Monocytes	5.4%
	Eosinophils	2.6%
	Basophils	0.1%
	RBC	4.24 × 10^6^/mm^3^
	Hemoglobin	11.8 g/dL
	Hematocrit	36.8%
	Platelets	246 × 10^3^/mm^3^
Arterial blood gas analysis		
	pH	7.401
	PaCO_2_	40.1 Torr
	PaO_2_	80.7 Torr
	HCO^3-^	24.4 mEq/L
Biochemistry		
	Total protein	6.6 g/dL
	Albumin	3.8 g/dL
	Total bilirubin	0.5 mg/dL
	Direct bilirubin	0.1 mg/dL
	AST	17 IU/L
	ALT	8 IU/L
	LDH	273 IU/L
	ALP	202 IU/L
	ChE	315 IU/L
	BUN	12.8 mg/dL
	Cr	0.5 mg/dL
	Na	140 mEq/L
	K	4.0 mEq/L
	Cl	106 mEq/L
	TC	193 mg/dL
	TG	175 mg/dL
Serology		
	CRP	<0.30 mg/dL
	KL-6	477U/mL
	SP-D	97.9 ng/mL
	ANA	(-)
	RF	<20U/mL
	Anti SS-A	<5.0U/mL
	Anti SS-B	<5.0U/mL
	Anti Jo-1	5.0U/mL
	PR3-ANCA	<10EU
	MPO-ANCA	<10EU
	*Mycoplasma*Ab	(-)
	*pneumoniae*IgG	(-)
	*C. pneumoniae* IgA	(-)
	β-d-Glucan	(-)
	CMV Ag (C7-HRP)	(-)
	DLST against imatinib	(-)

**Figure 2:**
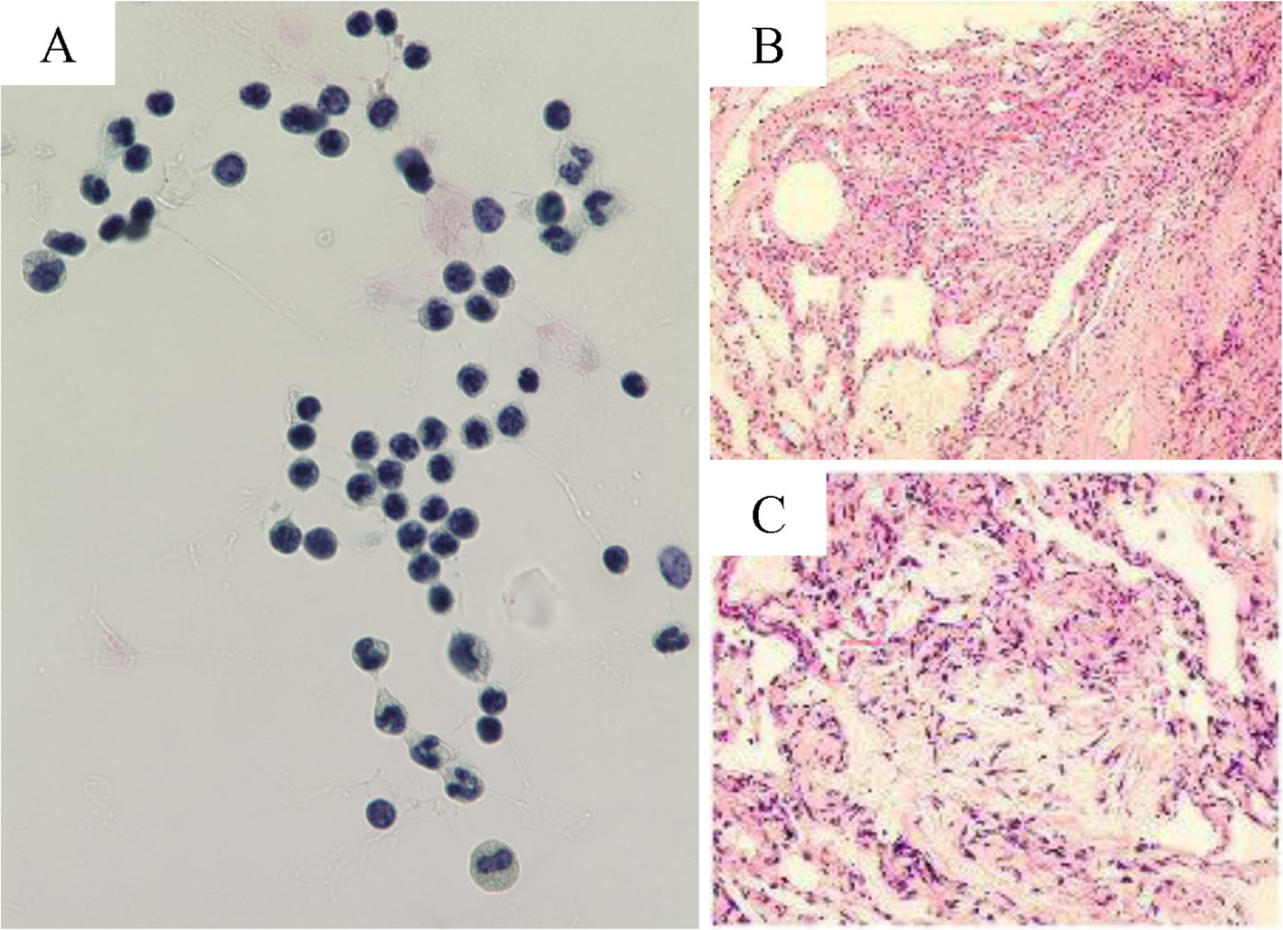
**Cytological and histopathological findings.** Cytological findings of BAL fluid (**A**) showed a large number of lymphocytes, but no malignancies. Lung specimens of the right B^2^b bronchus obtained by transbronchial lung biopsy (**B**, **C**) showed mixed intra-alveolar and interstitial changes, with loose fibrous plugs and giant cells in intra-alveolar regions, and fibroblasts and lymphocytes in interstitial tissue, which was suggestive of a hypersensitivity pneumonitis pattern.

Chest radiography on admission revealed bilateral reticular shadows in both mid and lower lung fields (Figure 1B). Chest high-resolution computed tomography (HRCT) scans revealed diffuse interstitial infiltrates in both lungs, predominantly along the bronchovascular bundles extending to subpleural regions (Figure 1D). Examination of bronchoalveolar lavage (BAL) fluid of the right B^4^ bronchus showed an elevated total cell count (4.35 × 10^5^ cells/mL) including 8% macrophages, 4% neutrophils, 71% lymphocytes, and 10% eosinophils. The CD4/CD8 ratio of lymphocyte subsets was 1.29 (Table 2). Cultures of BAL fluid were negative for fungal, bacterial, or mycobacterial pathogens. Cytological findings of BAL fluid showed a large number of lymphocytes, but no malignancies (Figure 2A). Lung specimens of the right B^2^b bronchus obtained by transbronchial lung biopsy showed mixed intra-alveolar and interstitial changes, with loose fibrous plugs and giant cells in intra-alveolar regions, and fibroblasts and lymphocytes in interstitial tissue, which was suggestive of a hypersensitivity pneumonitis pattern (Figure 2B, 2C).

**Table 2:** Findings of bronchoalveolar lavage fluid

Total cell counts	4.35 × 10^5^/mL
Macrophage	8%
Neutrophils	4%
Lymphocytes	71%
Eosinophils	10%
CD4/CD8 ratio	1.29

Although the result of DLST against imatinib was negative, drug-induced ILD caused by imatinib was suspected, because no other cause was evident. High-dose methylprednisolone (1 g/day intravenously for 3 days) was administered, followed by oral prednisolone (40 mg/day), which was then tapered off gradually. This resulted in an immediate improvement in clinical condition and a gradual improvement in chest radiographic findings (Figure 1C, 1E). She underwent total gastrectomy and partial hepatectomy for the treatment of GIST one year after the initiation of the corticosteroid treatment with no recurrence of ILD.

## Discussion

We encountered a unique case of imatinib-induced ILD, which developed after discontinuation of the drug. In addition to our report, 13 other case reports of imatinib-induced ILD have been published. All of these patients developed imatinib-induced ILD during administration of the drug (2 to 44 weeks), and in all cases, the condition was successfully treated with steroid therapy and drug discontinuation [5] (and references therein).

In one of these reports, Ohnishi et al. reported that 27 of 5,500 patients who were administered imatinib were diagnosed with drug-related ILD. Of the 27 patients with drug-induced ILD, 23 were diagnosed with CML and 4 with GIST. The median period until development of ILD was 49 days (range, 10–282 days). Results of DLST against imatinib were available for 9 patients, and all were negative. Imatinib-related ILD responded well to corticosteroid treatments [6].

Although imatinib was discontinued on the 73rd day of treatment, she developed fever, dry cough, and dyspnea on exertion 2 weeks after discontinuation of imatinib. HRCT scans revealed bilateral interstitial infiltrates predominantly along the bronchovascular bundles. Bronchoscopic examination showed no pathogens or malignancies. Although the result of a DLST against imatinib was negative, imatinib-induced ILD was the most probable diagnosis because no other cause was evident. Although uncommon, late-onset pneumonitis caused by cytotoxic anti-neoplastic drugs occasionally occurs even after discontinuation of the drugs. To the best of our knowledge, the present case is the first report of development of imatinib-induced ILD after discontinuation of the drug.

Imatinib plasma concentrations have been reported to increase by 2- to 3-fold when reaching steady state during 400 mg once-daily administration (to 2.6 ± 0.8 μg/mL at peak and 1.2 ± 0.8 μg/mL at trough), exceeding the 0.5 μg/mL (1 μmol/L) concentration required for tyrosine kinase inhibition in vitro. The terminal elimination half-life of imatinib is approximately 18 hours [7]. However, no evidence of tumor growth was noted in nude mice injected with the Bcr/Abl-positive human leukemia cell line KU812 and receiving imatinib at 160 mg/kg orally every 8 hours for 11 consecutive days, for up to 240 days [8]. Accordingly, the biological activity of imatinib may continue after administration has ceased; this may be one of the explanations for occurrence of drug-induced ILD after discontinuation of the drug. Another explanation can be derived from histopathological findings. A recent evidence supports the prominent role of T-helper 1 cell-mediated hypersensitivity with an imbalance of T-lymphocyte subsets in the late phase of hypersensitivity pneumonitis, although the deposition of immune complex may participate in the acute form of the disease as well as in the early phase of the chronic form [9] (and references therein). This delayed cell-mediated hypersensitivity mechanism may have played a role in the pathogenesis of the present case.

The proliferative activities of PDGFR and other tyrosine kinases in the pathogenesis of idiopathic pulmonary fibrosis led to in vivo and in vitro investigations to assess the use of imatinib as a potential inhibitor of lung fibrosis. Imatinib was identified as a potent inhibitor of lung fibroblast-to-myofibroblast transformation and proliferation as well as extracellular matrix production through inhibition of PDGF and transforming growth factor (TGF)-β signaling [10]. In addition, imatinib inhibits lung fibrosis in bleomycin models of lung fibrosis [11,12]. The interesting clinical question of why imatinib, which has an antifibrotic effect, causes drug-induced ILD remains to be answered in a future study.

## Conclusion

Pulmonary toxicities caused by targeted agents are rare but important to recognize even after discontinuation of the drug. We have reported a unique case of imatinib-induced ILD that developed after discontinuation of the drug. Physicians should consider the occurrence of imatinib-induced ILD even after discontinuation of the drug.

## Consent

Written informed consent was obtained from the patient for publication of this case report and any accompanying images. A copy of the written consent is available for review by the Editor-in-Chief of this journal.

## Abbreviations

BAL: Bronchoalveolar lavage; DDR: Discoidin domain receptors; DLST: Drug lymphocyte-stimulating test; GIST: Gastrointestinal stromal tumor; HRCT: High-resolution computed tomography; ILD: Interstitial lung disease; KL-6: Krebs von den Lungen-6; PDGFR: Platelet-derived growth factor receptor; SP-D: Surfactant protein-D; TGF: Transforming growth factor; TKI: Tyrosine kinase inhibitor.

## Competing interest

The authors declare that they have no competing interests.

## Authors’ contributions

TK and SN made substantial contributions to the study conception and design. TK and SN were involved in drafting the article and participated in the diagnosis and treatment of the lung disease of this patient. YN participated in the diagnosis and treatment of gastrointestinal stromal tumor of this patient. TK, HM, KH, ES and AK carried out the broncoscopy with bronchoalveolar lavage and transbronchial biopsy. NS, YI, HM and SK were involved in critically revising the article. All authors read and approved the final manuscript.
